# Outcome of Respiratory Distress in Neonates with Bubble CPAP at Neonatal Intensive Care Unit of a Tertiary Hospital

**DOI:** 10.31729/jnma.4294

**Published:** 2019-04-30

**Authors:** Sunil Raja Manandhar

**Affiliations:** 1 Department of Pediatrics, Kathmandu Medical College Teaching Hospital, Sinamangal, Nepal

**Keywords:** *bubble CPAP*, *neonates*, *respiratory distress*

## Abstract

**Introduction:**

Respiratory distress is one of the commonest problem seen in neonates during admission in Neonatal Intensive Care Unit. Hyaline Membrane disease, Meconium Aspiration Syndrome, septicemia, congenital pneumonia, Transient Tachypnea of Newborn are the major causes of respiratory distress in neonates. Bubble Continuous Positive Airway Pressure is a non-invasive respiratory support delivered to a spontaneously breathing newborn to maintain lung volume during expiration. The main objective of this study was to observe the outcome of respiratory distress in neonates with Bubble Continuous Positive Airway Pressure.

**Methods:**

This was a descriptive cross-sectional study conducted at Kathmandu Medical College Teaching Hospital over six months (October 2018 - March 2019) period. All preterm, term and post term babies with respiratory distress were included. Ethical clearance was received from Institutional Review Committee of Kathmandu Medical College and statistical analysis was done with SPSS 19 version.

**Results:**

Sixty three babies with respiratory distress were included in this study with 45 (71%) male predominance. The mean birth weight receiving Bubble Continuous Positive Airway Pressure was 2661.75+84 gms and gestational age was 36.67±3.4 wks. The Bubble Continuous Positive Airway Pressure was started at 8.05+2 hr of life and duration of Bubble Continuous Positive Airway Pressure required for settling respiratory distress was 95.71+3 hrs. Out of 63 babies, improvement of respiratory distress in neonates with Bubble Continuous Positive Airway Pressure was 39 (61%) with confidence interval of 38% to 62% whereas 24 (39%) babies required mechanical ventilation and other modalities.

**Conclusions:**

This study concludes usefulness of Bubble Continuous Positive Airway Pressure in neonates with respiratory distress.

## INTRODUCTION

Respiratory distress is one of the most common problem occurring; 5% in term and 29% in preterm babies requiring admission in Neonatal Intensive Care Unit (NICU) leading to 20% of neonatal deaths.^1–3^ Saeed Z et al. described respiratory distress as the most common presenting problem encountered within the first 48–72 hours of life with a prevalence of 4.24% in neonates.^4^ Hyaline Membrane Disease (HMD), Meconium Aspiration Syndrome (MAS), septicemia, congenital pneumonia, Transient Tachypnea of Newborn (TTN) are the major causes of respiratory distress in preterm, term and post term neonates.^5^

Continuous Positive Airway Pressure (CPAP) is an effective therapy for managing respiratory distress in preterm, term and post term neonates.^6^ CPAP is the application of positive pressure (4–6 cm of water) to the airways of spontaneously breathing babies throughout the respiratory cycle to reduce work of breathing, preventing lung collapse by maintaining functional residual capacity.^7–10^

The main objective of this study was to observe the outcome of respiratory distress in neonates with Bubble CPAP (B-CPAP).

## METHODS

This was a descriptive cross-sectional study done at 10 bedded NICU of Pediatrics Department, Kathmandu Medical College Teaching Hospital (KMCTH), Sinamangal, Kathmandu. Perinatal Mortality Rate (PMR) of this tertiary hospital is 10/1000 births and Neonatal mortality rate (NMR) is 4.5 /1000 live births.^11^ Ethical clearance was received from Institutional Review Committee (IRC) of Kathmandu Medical College and written consent was taken from parents after explaining the baby's condition requiring B-CPAP therapy. B-CPAP is a non-invasive ventilation to deliver CPAP in a spontaneously breathing newborn to maintain lung volumes during expiration.^12^ The study period was of six months (October 2018 to March 2019) duration. All preterm, term and post term babies born at KMCTH with respiratory distress due to any cause eg. Hyaline membrane disease (HMD), Meconium Aspiration Syndrome (MAS), septicemia, congenital pneumonia, Transient Tachypnea of Newborn (TTN) and perinatal asphyxia were included in this study. Syndromic babies and lethal congenital anomalies (eg. meningomyelocel, anencephaly, gastroschisis and diaphragmatic hernia) were excluded.

Sample size estimation:


$$\begin{array}{c} \text{s}= \end{array}\frac{{\text{Z}}^{2}}{}\frac{\times \text{p}\times \text{q}}{{\text{d}}^{2}}\begin{array}{c} =384 \end{array}$$

where,

s= calculated sample size for infinite population
n= sample size
Z=1.96 for Confidence Interval of 95%
p= prevalence of improvement of respiratory distress in neonates with B-CPAP, 50%
q = 1- p
N= definite population (70)
d = margin of error

considering 6% as a non-response rate, Total sample size was 63.

Sixty three neonates were included in this study and convenient sampling method was applied.

Respiratory distress was documented by fast breathing (Respiratory rate > 60/min) and followed by any one of the followings.^13^

1. Low O_2_ saturation (SPO_2_ < 87%)
2. Chest retraction
3. Grunting
4. Nasal flaring
5. Severe chest indrawing.

Severity of respiratory distress in neonates is assessed by Silverman's Anderson Respiratory Scoring. Silver-man's score 0 indicates no respiratory distress, 4–6 indicates moderate respiratory distress and 7–10 indicates severe respiratory distress. Silverman score > 4 were eligible for keeping babies under B-CPAP.^14^

Details of birth history, type of delivery along with maternal variables eg. multiple births, Pregnancy Induced Hypertension, Prolonged Rupture of Membrane (PROM) were recorded. Babies variables eg. birth weight, ges-tational age, Apgar score at 1minute and 5 minute, delivery room management (oxygen, tactile stimulation, bag and mask ventilation and intubation), silverman's scoring before and after starting B-CPAP were also recorded. Following parameters were observed during the study period: time of starting B-CPAP, respiratory rate before, during and after B-CPAP, total duration required to wean off B-CPAP, any case of B-CPAP failure requiring mechanical ventilation. The other clinical data recorded are Patent Ductus Arteriosus (PDA), Atrial Septal Defect (ASD) and Ventricular Septal Defect (VSD) (clinical and Echo proven), pneumothorax, culture positive sepsis, duration of hospital stay among the survivors. In this study, more term and normal birth weight babies could be studied, which could improve the outcome of B-CPAP. Statistical analysis was done with SPSS 19 version.

## RESULTS

There were total of 63 babies with respiratory distress included in this study. The mean birth weight of babies receiving B-CPAP was 2661.75±84 gms and mean gestational age was 36.67±3.4 weeks. Similarly, mean Silverman's score before starting B-CPAP was 5.03±1.13 and mean age of starting B-CPAP due to grunting and chest retraction was 8.05±26.03 hr of life. Total mean duration of B-CPAP required to settle respiratory distress in neonates with respiratory distress was 95.71±36.7 hrs. Mean duration of babies kept under mechanical ventilation was 15.06±42.1 hrs and mean hospital stay among the survivor neonates was 8±3.37 days (Table 1).

**Table 1:** Demographics and clinical parameters of babies under B-CPAP (n = 63).

S.N.	Variables	Mean	Range
1	Gestational Age	36.67±3.4	(28–41) wks.
2	Birth weight	2661.75±84	(800 −4500) gms
3	Silverman's score before starting Bubble CPAP	5.03±1.13	(4–8)
4	Mean age of Bubble CPAP started	8.05±26.03	(1–192 ) hrs
5	Apgar Score at 1 min	6.54±1.70	(3–8)
6.	Apgar Score at 5 min	7.89±1.30	(4–9)
7	Mothers Age	27.19±3.6	(19–38 ) yrs.
8	Mean duration of Maternal PROM (15 mothers)	37±24.2	(18–96) hrs
9	SPO2 in room air	84.73±2.16	(80–88) %
10	Total duration of Bubble CPAP required	95.71±36.7	(24–192) hrs
11	Total duration of Mechanical Ventilation required due to B-CPAP failure (12 babies)	15.06±42.1	(0–240)hrs
12	Total Hospital Stay (59 babies)	8±3.37	(3–15 )days

Forty five (71%) babies were term with male predominance 47 (75%). Out of 63 babies, 38 (60%) babies were of normal birth weight, regarding neonatal resuscitation, 42 (67%) babies did not require any form of resuscitation and only 4 (8%) babies required extensive neonatal resuscitation. The most common disease for starting B-CPAP was congenital pneumonia 15 (23%) followed by MAS 12 (19%) and Birth Asphyxia 8 (13%). There were 7 (11%) babies each had HMD and neonatal sepsis. Six babies (10%) were diagnosed as congenital acyanotic heart disease by Echo, out of which four had isolated ASD, one had ASD with VSD and one had ASD with PDA. During the study period, a total of 24 (39%) babies required additional treatment apart from B-CPAP. Twelve (19%) babies required mechanical ventilation due to B-CPAP failure and 4 (8%) babies with HMD required Surfactant Replacement Therapy (SRT). Similarly 6 (10%) babies received Frusemide for Echo proven severe pulmonary artery hypertension (PAH), 1 (2%) baby was treated with water seal drainage for right sided Pneumothorax and 1 (2%) baby required sodium bicarbonate correction for severe metabolic acidosis with neonatal sepsis. Out of 63 babies, 59 (94%) babies were survived and only 4 (6%) babies were expired. Prevalance of improved respiratory distress with B-CPAP was 39 (61%) at 95% confidence interval [38%-62%]. Three babies died due to septicemia with DIC and one baby died due to pulmonary hemorrhage. The commonest complication of B-CPAP seen in 10 (16%) neonates was superficial skin abrasion under the nose and over the cheeks. Four (6%) babies each had gastric distension and acquired secondary infection whereas 1 (2%) each had right sided pneumothorax and recurrent apnea respectively (Table 2).

**Table 2:** Neonatal Characteristics (n = 63).

S.N.	Variables	n	
1.	Baby sex		
	Male	47	(75)
	Female	16	(25)
	Total	63	(100)
2.	Gestation		
	Preterm	18	(29)
	Term	45	(71)
	Total	63	(100)
3.	Birth weight		
	Extremely low birth weight (<1kg)	2	(3)
	Very low birth weight (<1.5 kg)	8	(13)
	Low birth weight (<2.5 kg)	15	(24)
	Normal birth weight (2.5–4 kg)	38	(60)
	Total	63	(100)
4.	Resuscitation procedure done at birth		
	Not required	42	(67)
	Tactile stimulation	5	(7)
	Bag n Mask	12	(19)
	Bag n Mask and Chest Compression	1	(2)
	Bag n Mask, Chest Compression and Intubation	2	(3)
	Bag n Mask, Chest Compression, Intubation and drug	1 (	2)
	Total	63 (	100)
5.	Mode of delivery		
	Emergency cesarean section	41	(65)
	Elective cesarean section	9	(14)
	Normal delivery	13	(21)
	Total	63	(100)
6	Causes of Respiratory Distress requiring Bubble CPAP		
6.1	Congenital Pneumonia		
	o Maternal history of PROM	9	(14)
	o Maternal High Vaginal Swab (HVS) culture positive	3	(5)
	o Other	3	(5)
	Total	15	(23)
6.2	Meconium Aspiration Syndrome (MAS)	12	(19)
6.3	Birth Asphyxia		
	○ HIE stage I	2	(3)
	○ HIE stage II	6	(10)
	Total	8	(13)
6.4	Hyaline Membrane Disease (HMD)		
	○ Grade II	2	(3)
	○ Grade III	2	(3)
	○ Grade IV	3	(5)
	Total	7	(11)
6.5	Congenital Acyanotic Heart Disease		
	○ Atrial Septal Defect (ASD)	4	(6)
	○ ASD with Ventricular Septal Defect (VSD)	1	(2)
	○ ASD with PDA (Patent Ductus Arterious)	1	(2)
	Total	6	(10)
6.6	Neonatal Sepsis		
	○ Culture positive sepsis	4	(6)
	○ Culture negative sepsis	3	(5
	Total	7	(11)
6.7	Transient Tachypnea of Newborn (TTN)	6	(10)
6.8	Aspiration Pneumonia	2	(3)
	Total	63	(100)
7	Additional treatment required		
	Respiratory support by B-CPAP only	39	(61)
	Mechanical ventilation for B-CPAP failure	12	(19)
	Frusemide for severe Pulmonary artery hypertension (PAH)	6	(10)
	Surfactant Replacement Therapy (SRT) for HMD	4	(6)
	Water seal drainage for Pneumothorax	1	(2)
	Sodium bicarbonate correction for severe metabolic acidosis with neonatal sepsis	1	(2)
	Total	63	(100)
8	Outcome of babies under Bubble CPAP		
	Survived	59	(94)
	Expired	4	(6)
	Total	63	(100)
9	Cause of death		
	Pulmonary hemorrhage	1	(2)
	Septicemia with DIC	3	(5)
	Total	4	(6)
10	Complication of Bubble CPAP		
	No complication	43	(68)
	Superficial skin abrasion	10	(16)
	Secondary infection	4	(6)
	Gastric distension	4	(6)
	Right sided Pneumothorax	1	(2)
	Recurrent Apnea	1	(2)
	Total	63	(100)

The study highlighted that mean duration of B-CPAP use within 24 hrs was 94.47±36.9 hrs and after 24 hrs was 128±13.8 hrs (Table 3).

Before B-CPAP, the mean respiratory rate was 83.02±7/ min, which had decreased after 48–96 hrs of B-CPAP. Similarly, the initial PEEP required before B-CPAP was 5.63±0.6cm of water, which was decreased after 48–96 hrs of B-CPAP. It showed decreasing trend of respiratory rate and PEEP pressure requirement after the use of B-CPAP (Table 3).

**Table 3:** Time of starting B-CPAP with respect to its duration and time to wean.

	Age of starting B-CPAP		Duration of B-CPAP required		Time required to wean off from B-CPAP	
	Mean	Range	Mean	Range	Mean	Range
B-CPAP started within 24 hrs (n= 59)	2.83±2.6	(1–12) hrs	94.47±36.9	(24–192)hrs	4.28±2.02	(2–12)days
B-CPAP started after 24 hrs (n= 4)	85±73.7	(26–192)hrs	128 ±13.8	(120–144)hrs	6.75±2.8	(3–10)days

**Table 4:** Clinical parameters under B-CPAP.

	Respiratory Rate		PEEP requirement	
	Mean	Range	Mean	Range
Before B-CPAP	83.02 ± 7.4 (n= 63)	(68–110)/min	5.63±0.6 (n=63)	(4–7) cm of water
At 48 hrs of B-CPAP	73.23 ± 9.3 (n= 61)	(50–90) /min	4.98 ±0.7 (n= 61)	(4–7) cm of water
At 72 hrs of B-CPAP	63.75 ±9.8 (n= 53)	(46–86) /min	4.87 ±0.7 (n= 53)	(4–7) cm of water
At 96 hrs of B-CPAP	59.91 ±9.9 (n= 35)	(42–80) /min	4.51 ±0.7 (n= 35)	(4–7) cm of water

## DISCUSSION

This descriptive cross-sectional study included all newborns (preterm and term) with respiratory distress who were treated with B-CPAP as the primary respiratory support. The included newborns were mostly term (71%) and 60% babies were of normal weight (2.5–4 kg). The controversy of early vs delayed CPAP continues to have many trials favoring early CPAP were carried out to be better in the larger infants prior to routine use of antenatal steroids and postnatal surfactant.^15^ The evidence suggested exogenous surfactant use with early CPAP with brief ventilation in extremely Low Birth Weight (ELBW) babies had decreased the use of mechanical ventilation.^16^ Similarly, this study also showed B-CPAP within 24 hrs (early CPAP) in neonates with respiratory distress was effective in reducing respiratory effort and required lesser duration (94.47±36.9 hrs) of B-CPAP as compared to after 24 hrs (128±13.8 hrs). This finding was also strongly supported by the study done by Mathai SS et al, in which B-CPAP use within 24 hrs had less duration of B-CPAP (44.93±24.56 hrs) required as compared to after 24 hrs (85.57±54.16 hrs).^17^

This study described the commonest cause of respiratory distress requiring B-CPAP was congenital pneumonia (23%) followed by MAS (19%), birth asphyxia (13%) and HMD (11%). Mathur NB et al. postulated congenital pneumonia as the main cause of respiratory distress (68%) with maternal history of PROM >24 hrs as one of the risk factor for it.^18^ This study also corroborated similar findings as out of 15 babies with congenital pneumonia, nine babies had maternal history of PROM highlighting it as one of the major risk factor.

Lanieta et al successfully demonstrated the usefulness of B-CPAP in developing countries highlighting its cost effectiveness.^19^ Similarly in this study, out of 63 babies with B-CPAP 39 (61%) babies were improved.

Since, this is a single institutional study with convenient sampling, outcome might not be generalized. There are chances of selection bias.

## CONCLUSIONS

This study concludes usefulness of Bubble Continuous Positive Airway Pressure in neonates with respiratory distress.
